# *Yin-Cold* or* Yang-Heat* Syndrome Type of Traditional Chinese Medicine Was Associated with the Epidermal Growth Factor Receptor Gene Status in Non-Small Cell Lung Cancer Patients: Confirmation of a TCM Concept

**DOI:** 10.1155/2017/7063859

**Published:** 2017-01-22

**Authors:** Yan-juan Zhu, Hai-bo Zhang, Li-rong Liu, Yi-hong Liu, Fu-li Zhang, Jian-ping Bai, Yong Li, Yan-chun Qu, Xin Qu, Xian Chen, Yan Li, Shu-yi Luo, Ai-hua Ou

**Affiliations:** ^1^Oncology Department, Guangdong Provincial Hospital of Chinese Medicine, Guangzhou, China; ^2^Division of Speech & Hearing Sciences, The University of Hong Kong, Pokfulam, Hong Kong; ^3^Department of Big Medical Data, Guangdong Provincial Hospital of Chinese Medicine, Guangzhou, China

## Abstract

Traditional Chinese Medicine (TCM) therapies should be tailored according to the different syndrome types. In order to identify the relationship between the TCM* Yin-cold (YC)* or* Yang-heat (YH)* syndrome types and the* EGFR* gene status, we prospectively studied 310 NSCLC patients. TCM* YH* or* YC* was diagnosed by three TCM experts. TCM symptoms and signs were entered into a binary cluster analysis. The relationships between the* EGFR* gene status,* YH* or* YC* syndrome types, and classification by cluster analysis were analyzed using the chi-square test and multivariate logistic regression. In the 299 patients who had their* EGFR* gene tested, 45.24%* YC* (76/168) and 25.95%* YH* (34/131) patients had* EGFR* mutations (*p* = 0.001). Among the 292 patients entered into the cluster analysis, 132 were classified into group A, with signs and symptoms similar to* YC*, whereas 160 group B patients were similar to* YH*. In the 281 patients with* EGFR* tested, 45.67% group A (58/127) and 28.57% group B patients (44/154) had* EGFR* mutations (*p* = 0.003). The* EGFR* status was independently correlated with TCM syndrome type and classification by cluster analysis on multivariate logistic regression. NSCLC patients with* YC* were more likely to have* EGFR* gene mutations.

## 1. Introduction

Lung cancer is a leading cause of cancer mortality worldwide, more than 85% of which is non-small cell lung cancer (NSCLC) [1]. For the intermediate to advanced stage NSCLC patients with sensitive epidermal growth factor receptor* (EGFR)* gene mutation,* EGFR* tyrosine kinase inhibitor (*EGFR*-TKI) is the preferred strategy. However, even for the exclusively* EGFR*-mutant advanced NSCLC patients, the median progression-free survival (PFS) was only 9–13 months for those who accepted first-line* EGFR*-TKI therapy [2–5].

Traditional Chinese Medicine (TCM) has been widely used in at least 78 countries [6], especially in China. Many clinical trials have evaluated the efficacy of TCM therapies in combination with* EGFR*-TKIs [7–11]. However, most of them focused only on one special decoction or TCM patent prescription, without a universally accepted TCM syndrome type differentiation system. Even the TCM treatment principles in these studies were different. In the TCM theory, doctors should first differentiate the TCM syndrome types of the patients before deciding the therapy principles and medicine. Thus, we should first find out the distribution of the TCM syndrome types for the NSCLC patients before evaluating the efficacy of TCM therapies. However, the syndrome types in the TCM theory are too complicated. We need to differentiate not only the* Yin* and* Yang*,* cold* and* heat*, but also the* Interior* and* Exterior, Deficiency* and* Excess*, diseases in six meridians and twelve* zang-fu* organs, and sometimes diseases of* qi*,* blood*, and* body liquids*. This makes it hard to combine TCM with modern evidence based medicine (EBM).

In the famous TCM book entitled* Huangdi Neijing*, it is suggested that we should first differentiate the* Yin-cold (YC)* or* Yang-heat (YH)* type of the diseases. Some experts also believe that knowing the* YC *or* YH* types can guide the outline of TCM differentiation of the diseases [12]. Patients with* YC *syndrome type are more likely to have intolerance to cold, cough with thin or white sputum, anorexia, paler complexion, loose or watery stool, clear urine, fresh taste, weakness, paler tongue with teeth-prints at the tongue edge, white tongue coating, weak or light pulse, or other symptoms and signs with similar characteristics. Patients with* YH *syndrome type are more likely to have tolerance to cold or intolerance to heat, cough with thick and yellowish sputum, redder complexion, constipation or dry stool, dark urine, fidgeting or impatience, redder tongue, thick and yellow tongue coating, slippery or strong pulse, or other symptoms and signs with similar features. The* YC* or* YH* syndrome types are the two sides of a relative TCM concept. Not all of the listed symptoms or signs are necessary for the diagnosis of the* YC* or* YH*. They only help describe the typical features of patients with* YC* or* YH*, so that the diagnosis of a special patient's syndrome type can be decided by comparing his TCM characteristics with these typical features.

In the ISEL trial, before the identification of* EGFR* gene mutation, researchers found that gefitinib achieved better survival in female, nonsmoker patients with adenocarcinoma [13]. The same phenomenon was also noted in the BR.21 trial, where erlotinib was the study drug [14]. Meanwhile, Paez et al. [15] and Lynch et al. [16] found that activating somatic mutations of the* EGFR* gene was associated with response to EGFR-TKIs. Further clinical trials, especially the famous IPASS [17] and OPTIMAL [3] studies, confirmed that* EGFR* gene mutated patients achieved better PFS and response rate from gefitinib or erlotinib. Furthermore, in Paez et al.'s study [15], they found that* EGFR* mutations were more frequent in adenocarcinoma patients (21% versus 2%), in women (20% versus 9%), and in Japanese patients (26% versus 2%). In the IPASS [17] study population (patients from Asia, light, or nonsmokers and diagnosed with adenocarcinoma), 59.7% tumors had EGFR mutation, compared with the mutation rate of 12.1% in the unselected population of the ISEL study [13]. Therefore, clinicians usually consider the ethnic origin, smoking status, gender, and histologic findings to help identify patients who have a higher likelihood of having an* EGFR* mutation.

In the TCM theory, female patients are more likely to have* YC* compared to male patients. In addition, cigarettes are the source of toxic heat, so the nonsmokers would have* YC *characteristics. In addition, most of the patients taking* EGFR*-TKIs would have red acneiform rashes, which is a typical sign of* YH.* Therefore,* EGFR*-TKIs may have a* warming-Yang* effect in TCM theory and should be used in patients with* YC*. Thus, we put forward the hypothesis that patients with* YC* would be more likely to have* EGFR* gene mutations. In this study, we identified the relationship between the* YC* or* YH* and the* EGFR* gene status, which may be the theoretical basis for further studies combining TCM therapies with* EGFR*-TKIs in NSCLC patients.

## 2. Materials and Methods

### 2.1. Patients and EGFR Gene Test

We prospectively studied previously untreated patients with newly diagnosed NSCLC or those with recurrence but had received their last anticancer therapy >6 months from the diagnosis of recurrence. These patients were admitted to the Guangdong Lung Cancer Institute of Guangdong General Hospital from January to June 2014 or admitted to the Department of Oncology of Guangdong Provincial Hospital of Chinese Medicine from March 2013 to May 2015. Those with other types of uncontrolled malignancies, uncontrolled tubercular (TB) or other infections, underlying diseases that were severe or life threatening, or severe mental disorders were excluded from this study, because of the difficulty in differentiating their TCM syndrome types.

Patients who have had their cancer tissue* EGFR* gene status tested in other qualified hospitals only had their results recorded. Otherwise, the test for* EGFR* gene mutation in formalin-fixed, paraffin-embedded specimens was performed using the amplification refractory mutation system (ARMS) in the Department of Pathology of our hospital. All the patients provided written informed consent for this study and* EGFR* gene test. This study was approved by the Ethics Committee of Guangdong Provincial Hospital of Chinese Medicine.

### 2.2. TCM Syndrome Type Diagnosis

First, the TCM syndrome types,* YC* or* YH*, were diagnosed by the investigators. Meanwhile, a questionnaire that had 97 questions (106 for women) about symptoms and 54 questions about signs was also recorded (Supplementary Table 1 in Supplementary Material available online at https://doi.org/10.1155/2017/7063859). The questionnaire was formulated according to the textbook for TCM diagnostics (in Chinese), which includes the four classical diagnostic processes for TCM syndrome types: inspection, auscultation, inquiry, and palpation, so that can record the TCM characteristics. Second, another TCM expert, who was blinded to the former process, classified the patients as* YC* or* YH* according to his clinical experience and the answers recorded in the questionnaire. If the syndrome type diagnoses of these two experts were different, then a third TCM expert was invited to help with classifying a patient as* YC *or* YH*.

### 2.3. Statistical Analysis

First, the relationships between the TCM syndrome types (*YC *or* YH*) and the* EGFR* gene status (mutated or wild) were analyzed using the chi-square test (*p* < 0.05). Second, demographic and clinical characteristics were compared between the* YC* and* YH* groups. Those with significant difference between the two groups (*p* < 0.05), as well as the* EGFR* gene status, were entered into a multivariate logistic regression, using the forward stepwise method, to analyze the relation with the TCM syndrome types. Finally, considering the subjectivity of the TCM diagnosis, we carried out a binary cluster analysis. The symptoms and signs statistically correlated with the TCM syndrome types (*p* < 0.1), which appeared in ≥5% and ≤95% patients, were entered into the cluster analysis, dividing the patients into two groups. Then, the relationships between the TCM syndrome types, the* EGFR* status, and the classification by the cluster analysis were analyzed with the chi-square test (*p* < 0.05) and logistic regression. Data was documented using EpiData software (version 3.1) and analyzed using SPSS software (version 19.0).

## 3. Results

### 3.1. Patient Characteristics

A total of 310 patients were enrolled in our study. One hundred and seventy-five patients (56.45%) were* YC *according to the TCM theory, whereas the other 135 patients (43.55%) were* YH*. The demographic and clinical characteristics are shown in Table 1. More* YH* patients were male (82.09% versus 52.57%, *p* < 0.001) and had a history of smoking (75.56% versus 40.23%, *p* < 0.001). Patients in the* YH* group were older than those in the* YC* group (*p* = 0.028). Some cancer characteristics were also significantly different between the two groups. Patients with recurrence are more likely* YC* (16.57% versus 8.89%, *p* = 0.048). More of the* YC* patients had bone metastasis (48.47% versus 36.64%, *p* = 0.042) and nonsquamous cell pathology (89.66% versus 78.52%, *p* = 0.007). Notably, the mean blood pressure of* YH* patients was higher than* YC *patients (*p*⁡ = 0.026). There were no significant differences in ECOG performance status (PS) score, height, weight, primary location, stage, liver metastasis, lung metastasis, or brain metastasis between the two groups.

### 3.2. Relationship between EGFR Gene and TCM Syndrome Types

A total of 299 patients had their* EGFR* gene tested, including 121 tested by ARMS assay at our hospital, 134 tested by direct sequencing at Guangdong General Hospital, 10 tested at Sun Yat-sen University Cancer Center, 10 tested at the First Affiliated Hospital of Guangzhou Medical University, and 8 tested at Dian Diagnostics. All of these institutions are qualified for* EGFR* gene testing. The tissues of the other 13 patients were also tested in certified hospitals, including the First Clinical College of Southern Medical University, Cancer Center of Guangzhou Medical University, and the First Affiliated Hospital of Sun Yat-sen University. The test institutions for the three remaining patients were not recorded in our system. A total of 110 patients had* EGFR* mutations, including 51 with exon 19 deletion, 46 with exon 21 mutation, 9 with exon 20 mutation, and 3 with exon 18 mutation. Because this study started in 2013, when the detail of mutated sites of these exons did not yet attract our attention, we only recorded the mutated exons without details. However, 13 patients (8* YC *and 5* YH*) were known to have L858R mutation in exon 21, 1* (YC)* with L861Q in exon 21, 2* (YH)* with T790M in exon 20, and 2 (1* YC* and 1* YH*) with S768I in exon 20.* YC* patients were more likely to have the* EGFR* gene mutations than* YH *patients (45.24% versus 25.95%, *p* = 0.001, Table 1), as well as the exon 19 deletion (22.02% versus 10.77%, *p* = 0.011) and the exon 21 mutation (20.83% versus 8.46%, *p* = 0.004).

### 3.3. Logistic Regression

According to the chi-square test and the* t*-test, gender, age, smoke history, lung cancer history, bone metastasis, pathology, and mean blood pressure were significantly different between the* YC* and* YH* groups (Table 1), as well as the* EGFR* gene status. All of these demographic, clinical, and tumor characteristics were entered into a multivariate logistic regression, using the forward stepwise method. As a result, the* EGFR* gene status, as well as the mean blood pressure and smoke history, was independently associated with the TCM syndrome type (Table 2).

### 3.4. Cluster Analysis

Due to the subjectivity of the TCM diagnosis, most of the studies about TCM failed to provide a high quality of evidence. To minimize the influence of subjectivity on the diagnosis of* YC* or* YH* in our study, we carried out a binary cluster analysis. A total of 292 patients had complete data on the symptoms and signs needed, including the 11 patients who did not have their* EGFR* gene tested. They were entered into the binary cluster analysis, according to the recorded 25 symptoms and 26 signs statistically correlated with the TCM syndrome types (*p* < 0.1), which appeared in ≥5% and ≤95% patients, dividing the patients into two groups. The most common symptoms and signs of the 132 patients in group A were intolerance to cold, fresh taste, weakness, and teeth-prints at the tongue edge, which were typical of* YC*. Meanwhile, most of the rest 160 patients in group B experienced expectoration, thick and yellow tongue coating, dry lips, slippery pulse, and deep-colored urine, which were typical symptoms and signs for* YH* (Table 3). The classification by cluster analysis was also statistically associated with the TCM syndrome type, with 98 patients (74.24%) in group A and 66 (41.25%) in group B with* YC* (*p* < 0.001, Table 3). In addition, patients in group A, who were more likely to be* YC*, also had a higher chance carrying the mutated* EGFR* gene (45.67% versus 28.57%, *p* = 0.003, Table 3). On logistic regression analysis, the* EGFR* gene status, as well as the gender and mean blood pressure, was independently associated with the classification by the cluster analysis (Table 4).

## 4. Discussion

*EGFR*-TKIs are the preferred treatment for intermediate to advanced stage NSCLC in patients with* EGFR* gene mutation. However, the median PFS was only 9–13 months even for the exclusively* EGFR*-mutant advanced NSCLC patients [2–5, 18, 19]. TCM therapies have been widely used in cancer, including NSCLC. Although the reported trials combining TCM with* EGFR*-TKIs declared that their decoctions or patent prescriptions were effective, the TCM treatment principles of these decoctions or patent prescriptions were all different, or even opposite. For example, the TCM treatment principle of the decoction in Xiao et al.'s study [10] was strengthening vital* qi* and clearing cancer toxicity, especially clearing* heat*, whereas the principles in Yi-tang's study [11] included strengthening vital* qi*, warming-*Yang*, nourishing* Yin*, clearing* heat*, and activating blood stasis, etc. For the TCM patent prescriptions, Huachansu Injection in Jian's study [8] has the effect of clearing* Yang-heat,* whereas Kanglaite Injection in Qing-Hua and Gao-Feng's study [9] has the effect of warming*-Yang*. On the other hand, a case report by Hwang et al. reported that inappropriate TCM herbs induced resistance to gefitinib whereas withdrawing of the herbs caused sensitivity again to gefitinib [20]. As a result, although many studies have reported the efficacy of TCM combining with* EGFR*-TKIs, we still do not know how to choose the decoctions or patent prescriptions in clinical practice, because of lack of TCM treatment principles.

However, because of the complexity of TCM syndrome types, it is hard to form a widely accepted TCM syndrome type differentiation system. Because the* YC* or* YH* syndrome types can guide the outline of TCM differentiation of the diseases according to the traditional book* Huangdi Neijing* and modern studies [12], we believe that we should first differentiate these two types of TCM syndrome to guide the TCM treatment principles for NSCLC patients in combination with* EGFR*-TKIs. In our study, we have proved that* EGFR* gene mutation was associated with* YC*, according to both the univariate chi-square test and multivariate logistic regression. To reduce the subjectivity in TCM syndrome type diagnosis and differentiation, the* YC* or* YH* were differentiated according to three TCM experts' diagnosis. In addition, we carried out a cluster analysis to minimize the subjectivity of TCM diagnosis. As was shown in the results, the classification by cluster analysis also confirmed our hypothesis. Therefore, the association between the* YC* or* YH* and the* EGFR* gene status was credible.

Based on our initial finding, there are some TCM issues which warrant further research. First, although the* YC* or* YH* was significantly associated with the* EGFR* gene status, the correlation coefficient was only −0.198, and only 57.86% patients were exactly expected. In fact, diagnosing the different TCM syndrome types is very complicated. Aside from differentiating* Yin* and* Yang*,* cold* and* heat,* we also need to differentiate* Interior *and* Exterior*,* Deficiency *and* Excess*, diseases in six meridians and twelve* zang-fu *organs, and the different diseases of* qi*,* blood*, and* body liquids*. On the other hand, investigating the gene status of our NSCLC patients was also complicated, as they had* EGFR*,* ROS-1, ALK, RAS *and some other rare mutations. Therefore, there may be complex networks between the TCM syndrome types and the gene status, which has been studied in coronary heart disease [21]. We can further study the relationship between the complex TCM syndrome types and the complex gene status by next generation sequencing.

Secondly, because the* EGFR* gene mutated patients are more likely* YC* and the* EGFR*-TKIs may be effective for* warming-Yang*, the syndrome types should change during treatment with* EGFR*-TKIs, from* YC *to* YH*, according to the TCM theory. To prove the TCM efficacy of* EGFR*-TKIs, we need to compare the TCM syndrome types of patients before and after taking* EGFR*-TKIs, especially during disease progression. Finally, the most common side effect of* EGFR*-TKIs was red acneiform rashes [2–5, 18, 19], with thirsty, red, and dry tongue and yellow tongue coating, which are also the evidence for the* warming-Yang* influence of* EGFR*-TKIs in TCM theory. However, we still do not know whether the* warming-Yang *influence of* EGFR*-TKIs is a treatment effect or just a kind of side effect. If the* warming-Yang* influence is a therapeutic effect, then patients with* YH* should achieve better prognosis, and TCM therapies with* warming-Yang* principles may help to improve the efficacy of* EGFR*-TKIs. By comparing the PFS and overall survival (OS) of the patients with* YC* or* YH *at diagnosis and progression after taking* EGFR*-TKIs, we can also find out the TCM treatment principles to delay the resistance to* EGFR*-TKIs. We will report the results as soon as the survival data is mature. All this research will be meaningful for guiding the principle of TCM therapies in combination with* EGFR*-TKIs, because we have proved the relationship of* YC *or* YH* with* EGFR* gene status.

## 5. Conclusions

In conclusion, we found that patients with YC have a higher chance with* EGFR* gene mutation. The influences on PFS and OS of TCM syndrome types (*YC* or* YH*) and TCM treatment principles (*warming-Yang* or* clearing-heating*) warrant further study, based on the confirmation of our TCM concept.

## Supplementary Material

Supplementary Table 1: the questionnaire is used to record the TCM characteristics.

## Figures and Tables

**Table 1:** Demographic, clinical, and tumoral characteristics of 310 patients.

Characteristics	Total (*n* = 310)	YC (*n* = 175)	YH (*n* = 135)	*p*
	(11 without EGFR status)	(7 without EGFR status)	(4 without EGFR status)	
Gender				<0.001^*∗*^
Male (*n*, %)	202 (65.37%)	92 (52.57%)	110 (82.09%)	
Female (*n*, %)	107 (34.63%)	83 (47.43%)	24 (17.91%)	
Age (year, mean ± SD)	60.67 ± 10.86	59.78 ± 11.48	61.83 ± 9.90	0.028^&^
Smoker				<0.001^*∗*^
Yes (*n*, %)	172 (55.66%)	70 (40.23%)	102 (75.56%)	
No (*n*, %)	137 (44.34%)	104 (59.77%)	33 (24.44%)	
Cancer history				0.048^*∗*^
Newly diagnosed (*n*, %)	269 (86.77%)	146 (83.43%)	123 (91.11%)	
Recurrence (*n*, %)	41 (13.23%)	29 (16.57%)	12 (8.89%)	
Primary location				0.069^*∗*^
Peripheral (*n*, %)	211 (73.26%)	124 (77.5%)	87 (67.97%)	
Central (*n*, %)	77 (26.74%)	36 (22.5%)	41 (32.03%)	
Stage				0.054^*∗*^
Non-IV (*n*, %)	69 (22.40%)	32 (18.39%)	37 (27.61%)	
IV (*n*, %)	239 (77.60%)	142 (81.61%)	97 (72.39%)	
Lung metastasis				0.091^*∗*^
Yes (*n*, %)	92 (30.16%)	58 (34.12%)	34 (25.19%)	
No (*n*, %)	213 (69.84%)	112 (65.88%)	101 (74.81%)	
Bone metastasis				0.042^*∗*^
Yes (*n*, %)	127 (43.20%)	79 (48.47%)	48 (36.64%)	
No (*n*, %)	167 (56.80%)	84 (51.53%)	83 (63.36%)	
Liver metastasis				0.562^*∗*^
Yes (*n*, %)	26 (8.64%)	16 (9.47%)	10 (7.58%)	
No (*n*, %)	275 (91.36%)	153 (90.53%)	122 (92.42%)	
Brain metastasis				0.493^*∗*^
Yes (*n*, %)	60 (20.27%)	36 (21.69%)	24 (18.46%)	
No (*n*, %)	236 (79.73%)	130 (78.31%)	106 (81.54%)	
Pathology				0.007^*∗*^
Nonsquamous (*n*, %)	262 (84.79%)	156 (89.66%)	106 (78.52%)	
Squamous (*n*, %)	47 (15.21%)	18 (10.34%)	29 (21.48%)	
PS				0.472^*∗*^
0~1	260 (85.53%)	151 (86.78%)	109 (83.85%)	
2~4	44 (14.47%)	23 (13.22%)	21 (16.15%)	
Height (cm, mean ± SD)	163.29 ± 7.57	161.14 ± 7.45	166.17 ± 6.76	0.074^&^
Weight (kg, mean ± SD)	59.40 ± 10.69	58.00 ± 10.91	61.27 ± 10.14	0.445^&^
Mean BP (mmHg, mean ± SD)	92.56 ± 10.16	91.15 ± 9.49	94.38 ± 10.72	0.026^&^
EGFR status *※*				0.001^*∗*^
Mutated (*n*, %)	110 (36.79%)	76 (45.24%)	34 (25.95%)	
Wild (*n*, %)	189 (63.21%)	92 (54.76%)	97 (74.05%)	
Exon 19 deletion				0.011^*∗*^
Yes (*n*, %)	51 (17.11%)	37 (22.02%)	14 (10.77%)	
No (*n*, %)	247 (82.89%)	131 (77.98%)	116 (89.23%)	
Exon 21 mutation				0.004^*∗*^
Yes (*n*, %)	46 (15.44%)	35 (20.83%)	11 (8.46%)	
No (*n*, %)	252 (84.56%)	133 (79.17%)	119 (91.54%)	
Rare mutation				0.097^*∗*^
Yes (*n*, %)	12 (4.03%)	4 (2.38%)	8 (6.15%)	
No (*n*, %)	286 (95.97%)	164 (97.62%)	122 (93.85%)	

^*∗*^Chi-square test.

^&^ *t*-test.

^*※*^Spearman correlation coefficient: −0.198, *p* = 0.001 (TCM syndrome types: Yin-cold = 0, Yang-heat = 1; EGFR status: wild = 0, mutated = 1).

**Table 2:** Logistic regression for TCM syndrome types, YC = 0 and YH = 1; *n* = 276.

Variables	*β*	SE	Exp(*β*)	*p*
BP	0.040	0.014	1.041	0.003
Smoker (yes = 1, no = 0)	1.612	0.287	5.013	<0.001
EGFR (mutated = 1, wild = 0)	−0.609	0.304	0.544	0.045
Constant	−4.720	1.298	0.009	<0.001

Adjusted by age, pathology, gender, lung cancer history, and bone metastasis.

**Table 3:** Clinical characteristics of 292 patients classified by the cluster analysis.

	Group A (*n* = 132)	Group B (*n* = 160)	*p*
Symptoms and signs (*n* = 292)			
Intolerance of cold	81 (61.4%)	52 (32.5%)	<0.001
Fresh taste	80 (60.6%)	60 (37.5%)	<0.001
Weakness	78 (59.1%)	65 (40.6%)	0.002
Teeth-prints at the tongue edge	76 (57.6%)	53 (33.1%)	<0.001
Expectoration	76 (57.6%)	113 (70.6%)	0.020
Yellow tongue coating	52 (39.4%)	110 (68.8%)	<0.001
Thick tongue coating	54 (40.9%)	102 (63.8%)	<0.001
Dry lips	63 (47.7%)	102 (63.8%)	0.006
Slippery pulse	25 (18.9%)	97 (60.6%)	<0.001
Deep-colored urine	74 (56.1%)	90 (56.3%)	0.974
Red tongue	31 (23.5%)	86 (53.8%)	<0.001
TCM syndrome type (*n* = 292)^*∗*^			
Yin-cold	98 (74.24%)	66 (41.25%)	<0.001
Yang-heat	34 (25.76%)	94 (58.75%)	
EGFR gene status (*n* = 281)^*∗∗*^			
Wild type	69 (54.33%)	110 (71.43%)	0.003
Mutated type	58 (45.67%)	44 (28.57%)	

^*∗*^ Spearman correlation coefficient: 0.331, *p* < 0.001. Group A = 0 and group B = 1; *Yin-cold* = 0, *Yang-heat* = 1.

^*∗∗*^ Spearman correlation coefficient: −0.177, *p* = 0.003. Group A = 0 and group B = 1; wild = 0, mutated = 1.

**Table 4:** Logistic regression for classification by cluster analysis, group A = 0 and group B = 1; *n* = 259.

Variables	*β*	SE	Exp(*β*)	*p*
BP	0.038	0.014	1.04	0.007
Gender (male = 1, female = 0)	1.285	0.298	3.615	<0.001
EGFR (mutated = 1, wild = 0)	−0.667	0.303	0.513	0.028
Constant	−1.418	1.339	0.242	0.289

Adjusted by age, pathology, smoke history, lung cancer history, and bone metastasis.
