# Supplementary material for: Yin-Cold or Yang-Heat Syndrome Type of Traditional Chinese Medicine Was Associated with the Epidermal Growth Factor Receptor Gene Status in Non-Small Cell Lung Cancer Patients: Confirmation of a TCM Concept
Source: Evid Based Complement Alternat Med. 2017 Jan 22;2017:7063859. doi: 10.1155/2017/7063859 (PMC5292165; doi:10.1155/2017/7063859)
Supplement: Supplementary file 1 — Supplementary Table 1: the questionnaire is used to record the TCM characteristics. [file 7063859.f1.doc]

Supplementary table 1. The questionnaire used to record the TCM characteristics

| Questions | Answers |
| --- | --- |
| Inquiry (symptoms) | |
| 1.Fatigue or weakness | 0.No 1.A little 2.Moderately  3.Quite a bit 4. Extremely |
| 2.Shortness of breath | 0.No 1.A little 2.Moderately  3.Quite a bit 4. Extremely |
| 3.Palpitation | 0.No 1.A little 2.Moderately  3.Quite a bit 4. Extremely |
| 4.Fidgeting or impatience | 0.No 1.A little 2.Moderately  3.Quite a bit 4. Extremely |
| 5.Frequent sighing | 0.No 1.A little 2.Moderately  3.Quite a bit 4. Extremely |
| 6.Poor memory | 0.No 1.A little 2.Moderately  3.Quite a bit 4. Extremely |
| 7.Uncommunicative or quietness preferred | 0.No 1.A little 2.Moderately  3.Quite a bit 4. Extremely |
| 8.Intolerance to cold | 0.No 1.A little 2.Moderately  3.Quite a bit 4. Extremely |
| 9. Often feeling cold at the hands and foots | 0.No 1.A little 2.Moderately  3.Quite a bit 4. Extremely |
| 10. Often feeling cold at the abdomen | 0.No 1.A little 2.Moderately  3.Quite a bit 4. Extremely |
| 11. Often feeling cold at the back, waist or knees | 0.No 1.A little 2.Moderately  3.Quite a bit 4. Extremely |
| 12. Often feeling hot (a feeling, not fever) | 0.No 1.A little 2.Moderately  3.Quite a bit 4. Extremely |
| 13. Often feeling hot at the face | 0.No 1.A little 2.Moderately  3.Quite a bit 4. Extremely |
| 14. Often feeling hot at the hands and foots | 0.No 1.A little 2.Moderately  3.Quite a bit 4. Extremely |
| 15. Often feeling hot at the thorax cavity | 0.No 1.A little 2.Moderately  3.Quite a bit 4. Extremely |
| 16. Often feeling hot at the stomach and esophagus | 0.No 1.A little 2.Moderately  3.Quite a bit 4. Extremely |
| 17. Often feeling sudden hot | 0.No 1.A little 2.Moderately  3.Quite a bit 4. Extremely |
| 18. Often feeling hot at a regular time everyday | 0.No 1.A little 2.Moderately  3.Quite a bit 4. Extremely |
| 19. Often feeling cold and hot alternately | 0.No 1.A little 2.Moderately  3.Quite a bit 4. Extremely |
| 20. Preferring warm or hot food or intolerance to cold food | 0.No 1.A little 2.Moderately  3.Quite a bit 4. Extremely |
| 21. Preferring cold or cool food or intolerance to hot food | 0.No 1.A little 2.Moderately  3.Quite a bit 4. Extremely |
| 22. Often feeling thirsty | 0.No 1.A little 2.Moderately  3.Quite a bit 4. Extremely |
| 23. Little drinking even thirsty | 0.No 1.A little 2.Moderately  3.Quite a bit 4. Extremely |
| 24. Preferring hot or warm water if thirsty | 0.No 1.A little 2.Moderately  3.Quite a bit 4. Extremely |
| 25. Preferring cold or cool water if thirsty | 0.No 1.A little 2.Moderately  3.Quite a bit 4. Extremely |
| 26.Bitter taste | 0.No 1.A little 2.Moderately  3.Quite a bit 4. Extremely |
| 27.Fresh taste | 0.No 1.A little 2.Moderately  3.Quite a bit 4. Extremely |
| 28.Bad breath out of mouth | 0.No 1.A little 2.Moderately  3.Quite a bit 4. Extremely |
| 29. Thick and viscous saliva | 0.No 1.A little 2.Moderately  3.Quite a bit 4. Extremely |
| 30. Often feeling thirsty and dry at the throat | 0.No 1.A little 2.Moderately  3.Quite a bit 4. Extremely |
| 31. Often feeling dry and hot at the nasal cavity | 0.No 1.A little 2.Moderately  3.Quite a bit 4. Extremely |
| 32. Often feeling pain and hot at the throat | 0.No 1.A little 2.Moderately  3.Quite a bit 4. Extremely |
| 33. Often feeling pain and hot at the gum | 0.No 1.A little 2.Moderately  3.Quite a bit 4. Extremely |
| 34. Repeated ulcer in mouth | 0.No 1.A little 2.Moderately  3.Quite a bit 4. Extremely |
| 35. Often feeling dry of eyes | 0.No 1.A little 2.Moderately  3.Quite a bit 4. Extremely |
| 36. Often with conjunctive redness | 0.No 1.A little 2.Moderately  3.Quite a bit 4. Extremely |
| 37. Often sweating even without or with light movement | 0.No 1.A little 2.Moderately  3.Quite a bit 4. Extremely |
| 38. Often sweating during sleeping | 0.No 1.A little 2.Moderately  3.Quite a bit 4. Extremely |
| 39. Often sweating only at the head | 0.No 1.A little 2.Moderately  3.Quite a bit 4. Extremely |
| 40. Often sweating only at one side of the body | 0.No 1.A little 2.Moderately  3.Quite a bit 4. Extremely |
| 41. Often sweating only at the chest | 0.No 1.A little 2.Moderately  3.Quite a bit 4. Extremely |
| 42. Often sweating at the hands and foots | 0.No 1.A little 2.Moderately  3.Quite a bit 4. Extremely |
| 43. Constipation or dry stool | 0.No 1.A little 2.Moderately  3.Quite a bit 4. Extremely |
| 44. Diarrhea or loose, watery stool | 0.No 1.A little 2.Moderately  3.Quite a bit 4. Extremely |
| 45. What’s the color of the stool | A. didn’t notice  B. Brown or similar  C. Yellow or similar  D. Mucous or purulent  E. Other, please describe |
| 46. Hematochezia | 0.No 1.A little 2.Moderately  3.Quite a bit 4. Extremely |
| 47. Often feeling inside tenesmus after defecating | 0.No 1.A little 2.Moderately  3.Quite a bit 4. Extremely |
| 48. Often feeling pain or burning when defecating | 0.No 1.A little 2.Moderately  3.Quite a bit 4. Extremely |
| 49. Often lack of strength for defecating | 0.No 1.A little 2.Moderately  3.Quite a bit 4. Extremely |
| 50. Often with dark urina sanguinis | 0.No 1.A little 2.Moderately  3.Quite a bit 4. Extremely |
| 51. Often with dark urine | 0.No 1.A little 2.Moderately  3.Quite a bit 4. Extremely |
| 52. Often feeling pain or burning when urinating | 0.No 1.A little 2.Moderately  3.Quite a bit 4. Extremely |
| 53. Urinating more often than normal | 0.No 1.A little 2.Moderately  3.Quite a bit 4. Extremely |
| 54. Urinating more often during night than normal | 0.No 1.A little 2.Moderately  3.Quite a bit 4. Extremely |
| 55. Cough | 0.No 1.A little 2.Moderately  3.Quite a bit 4. Extremely |
| 56. Do you cough more in daytime or night? | A. No different  B. In daytime  C. In night |
| 57. Expectoration | 0.No 1.A little 2.Moderately  3.Quite a bit 4. Extremely |
| 58. Yellowish sputum | 0.No 1.A little 2.Moderately  3.Quite a bit 4. Extremely |
| 59. Please describe the sputum | A. No expectoration  B. loose or watery sputum  C. Frothy sputum  D. Thick and excessive sputum  E. Thick but little sputum  F. Other, please describe |
| 60. Hemoptysis | 0.No 1.A little 2.Moderately  3.Quite a bit 4. Extremely |
| 61. What’s the color of your bloody sputum? | A. No hemoptysis  B. Light red  C. Bright red  D. Dull red |
| 62. With blood clots in your bloody sputum | A. No hemoptysis  B. Yes C. No |
| 63. Chest tightness | 0.No 1.A little 2.Moderately  3.Quite a bit 4. Extremely |
| 64. Chest pain | 0.No 1.A little 2.Moderately  3.Quite a bit 4. Extremely |
| 65. Relief of chest pain after cold or cool compress | 0.No 1.A little 2.Moderately  3.Quite a bit 4. Extremely |
| 66. Relief of chest pain after hot or warm compress | 0.No 1.A little 2.Moderately  3.Quite a bit 4. Extremely |
| 67. Please describe the feeling of chest pain | B. Pain with distension  C. Piercing pain  D. Colic pain  E. Cutting pain  F. Burning pain  G. Other, please describe |
| 68. Anorexia | 0.No 1.A little 2.Moderately  3.Quite a bit 4. Extremely |
| 69. Nausea and vomiting | 0.No 1.A little 2.Moderately  3.Quite a bit 4. Extremely |
| 70. Often feeling hungry or even after just eating | 0.No 1.A little 2.Moderately  3.Quite a bit 4. Extremely |
| 71. Flatulence in gastric | 0.No 1.A little 2.Moderately  3.Quite a bit 4. Extremely |
| 72. Abdominal Pain | 0.No 1.A little 2.Moderately  3.Quite a bit 4. Extremely |
| 73. Relief of abdominal pain after cold or cool compress | 0.No 1.A little 2.Moderately  3.Quite a bit 4. Extremely |
| 74. Relief of abdominal pain after hot or warm compress | 0.No 1.A little 2.Moderately  3.Quite a bit 4. Extremely |
| 75. Please describe the feeling of abdominal pain | B. Pain with distension  C. Piercing pain  D. Colic pain  E. Cutting pain  F. Burning pain  G. Other, please describe |
| 76. Difficulty in sleeping | 0.No 1.A little 2.Moderately  3.Quite a bit 4. Extremely |
| 77. Much dreams or nightmare | 0.No 1.A little 2.Moderately  3.Quite a bit 4. Extremely |
| 78. Lassitude or fatigue in waist or legs | 0.No 1.A little 2.Moderately  3.Quite a bit 4. Extremely |
| 79. Dizziness | 0.No 1.A little 2.Moderately  3.Quite a bit 4. Extremely |
| 80. Tinnitus | 0.No 1.A little 2.Moderately  3.Quite a bit 4. Extremely |
| 81. Please describe the sound of the tinnitus | A. No tinnitus  B. Ringing tinnitus  C. Thundering tinnitus  D. Other, please describe |
| 82. Feeling of numbness | 0.No 1.A little 2.Moderately  3.Quite a bit 4. Extremely |
| 83. Headache | 0.No 1.A little 2.Moderately  3.Quite a bit 4. Extremely |
| 84. Relief of headache after cold or cool compress | 0.No 1.A little 2.Moderately  3.Quite a bit 4. Extremely |
| 85. Relief of headache after hot or warm compress | 0.No 1.A little 2.Moderately  3.Quite a bit 4. Extremely |
| 86. Please describe the feeling of headache | B. Pain with distension  C. Piercing pain  D. Colic pain  E. Cutting pain  F. Burning pain  G. Other, please describe |
| 87. Backache | 0.No 1.A little 2.Moderately  3.Quite a bit 4. Extremely |
| 88. Relief of backache after cold or cool compress | 0.No 1.A little 2.Moderately  3.Quite a bit 4. Extremely |
| 89. Relief of backache after hot or warm compress | 0.No 1.A little 2.Moderately  3.Quite a bit 4. Extremely |
| 90. Please describe the feeling of backache | B. Pain with distension  C. Piercing pain  D. Colic pain  E. Cutting pain  F. Burning pain  G. Other, please describe |
| 91. Arthralgia or bone pain | 0.No 1.A little 2.Moderately  3.Quite a bit 4. Extremely |
| 92. Relief of arthralgia after cold or cool compress | 0.No 1.A little 2.Moderately  3.Quite a bit 4. Extremely |
| 93. Relief of arthralgia after hot or warm compress | 0.No 1.A little 2.Moderately  3.Quite a bit 4. Extremely |
| 94. Please describe the feeling of arthralgia | B. Pain with distension  C. Piercing pain  D. Colic pain  E. Cutting pain  F. Burning pain  G. Other, please describe |
| 95.Dystension or pain at the sides of the chest or at the breasts | 0.No 1.A little 2.Moderately  3.Quite a bit 4. Extremely |
| 96.Unclear vision | 0.No 1.A little 2.Moderately  3.Quite a bit 4. Extremely |
| 97. Petechiae or ecchymosis even without hurt or injury | 0.No 1.A little 2.Moderately  3.Quite a bit 4. Extremely |
| For women only | |
| 1. Yellowish leucorrhea | 0.No 1.A little 2.Moderately  3.Quite a bit 4. Extremely |
| 2. Thick leucorrhea | 0.No 1.A little 2.Moderately  3.Quite a bit 4. Extremely |
| 3. Smelly leucorrhea | 0.No 1.A little 2.Moderately  3.Quite a bit 4. Extremely |
| For pre-menopausal women only | |
| 1. Dysmenorrhea | 0.No 1.A little 2.Moderately  3.Quite a bit 4. Extremely |
| 2. Relief of dysmenorrheal after hot or warm compress | 0.No 1.A little 2.Moderately  3.Quite a bit 4. Extremely |
| 3. Hypermenorrhea | 0.No 1.A little 2.Moderately  3.Quite a bit 4. Extremely |
| 4. Hypomenorrhea | 0.No 1.A little 2.Moderately  3.Quite a bit 4. Extremely |
| 5. Dull and dark menstruation | 0.No 1.A little 2.Moderately  3.Quite a bit 4. Extremely |
| 6. With blood clots in your menstruation | 0.No 1.A little 2.Moderately  3.Quite a bit 4. Extremely |
| Inspection (signs) | |
| 1. Silent or Depressed | 0.No 1.A little 2.Moderately  3.Quite a bit 4. Extremely |
| 2. Active or lively | 0.No 1.A little 2.Moderately  3.Quite a bit 4. Extremely |
| 3. Out of spirits | 0.No 1.A little 2.Moderately  3.Quite a bit 4. Extremely |
| 4. Redder complexion | 0.No 1.A little 2.Moderately  3.Quite a bit 4. Extremely |
| 5. Paler complexion | 0.No 1.A little 2.Moderately  3.Quite a bit 4. Extremely |
| 6. Darker or gloomy complexion | 0.No 1.A little 2.Moderately  3.Quite a bit 4. Extremely |
| 7. Sallow complexion | 0.No 1.A little 2.Moderately  3.Quite a bit 4. Extremely |
| 8. Dim and swollen complexion | 0.No 1.A little 2.Moderately  3.Quite a bit 4. Extremely |
| 9. Redder lips | 0.No 1.A little 2.Moderately  3.Quite a bit 4. Extremely |
| 10. Paler lips | 0.No 1.A little 2.Moderately  3.Quite a bit 4. Extremely |
| 11. Darker or gloomy lips | 0.No 1.A little 2.Moderately  3.Quite a bit 4. Extremely |
| 12. Dry lips | 0.No 1.A little 2.Moderately  3.Quite a bit 4. Extremely |
| 13. Oily at the face | 0.No 1.A little 2.Moderately  3.Quite a bit 4. Extremely |
| 14. Acne | 0.No 1.A little 2.Moderately  3.Quite a bit 4. Extremely |
| 15. Flushing at the cheeks | 0.No 1.A little 2.Moderately  3.Quite a bit 4. Extremely |
| 16. Dark rim of the eyes | 0.No 1.A little 2.Moderately  3.Quite a bit 4. Extremely |
| 17. Rough skin | 0.No 1.A little 2.Moderately  3.Quite a bit 4. Extremely |
| Tongue Inspection | |
| 1. Redder tongue | 0.No 1.A little 2.Moderately  3.Quite a bit 4. Extremely |
| 2. Paler tongue | 0.No 1.A little 2.Moderately  3.Quite a bit 4. Extremely |
| 3. Redder tongue tip | 0.No 1.A little 2.Moderately  3.Quite a bit 4. Extremely |
| 4. Darker or gloomy tongue | 0.No 1.A little 2.Moderately  3.Quite a bit 4. Extremely |
| 5. Plump tongue | 0.No 1.A little 2.Moderately  3.Quite a bit 4. Extremely |
| 6. Thin tongue | 0.No 1.A little 2.Moderately  3.Quite a bit 4. Extremely |
| 7. Teeth-prints at tongue edge | 0.No 1.A little 2.Moderately  3.Quite a bit 4. Extremely |
| 8. Tough tongue | 0.No 1.A little 2.Moderately  3.Quite a bit 4. Extremely |
| 9. Tender tongue | 0.No 1.A little 2.Moderately  3.Quite a bit 4. Extremely |
| 10. Tongue with red spots | 0.No 1.A little 2.Moderately  3.Quite a bit 4. Extremely |
| 11. Tongue with petechiae or ecchymosis | 0.No 1.A little 2.Moderately  3.Quite a bit 4. Extremely |
| 12. Tongue with fissure or crack | 0.No 1.A little 2.Moderately  3.Quite a bit 4. Extremely |
| 13. Varicose and dark sublingual vein | 0.No 1.A little 2.Moderately  3.Quite a bit 4. Extremely |
| 14. Thicker and heavier tongue coating | 0.No 1.A little 2.Moderately  3.Quite a bit 4. Extremely |
| 15. Thinner and less tongue coating | 0.No 1.A little 2.Moderately  3.Quite a bit 4. Extremely |
| 16. Yellow tongue coating | 0.No 1.A little 2.Moderately  3.Quite a bit 4. Extremely |
| 17. Black or brown tongue coating | 0.No 1.A little 2.Moderately  3.Quite a bit 4. Extremely |
| 18. Dry tongue coating | 0.No 1.A little 2.Moderately  3.Quite a bit 4. Extremely |
| 19. Moist tongue coating | 0.No 1.A little 2.Moderately  3.Quite a bit 4. Extremely |
| 20. Rough tongue coating | 0.No 1.A little 2.Moderately  3.Quite a bit 4. Extremely |
| Auscultation (signs) | |
| 1. Weak and lack of strength to cough | 0.No 1.A little 2.Moderately  3.Quite a bit 4. Extremely |
| 2. Weak and lack of strength to speak | 0.No 1.A little 2.Moderately  3.Quite a bit 4. Extremely |
| 3. Ponderous breathing | 0.No 1.A little 2.Moderately  3.Quite a bit 4. Extremely |
| Palpation (signs) | |
| 1. Colder skin temperature at the hands and foods (felt by the investigator) | 0.No 1.A little 2.Moderately  3.Quite a bit 4. Extremely |
| 2. Heated skin temperature at the hands and foods (felt by the investigator) | 0.No 1.A little 2.Moderately  3.Quite a bit 4. Extremely |
| 3. Swelling at the limbs | 0.No 1.A little 2.Moderately  3.Quite a bit 4. Extremely |
| 4. Redder skin at the swollen limbs | 0.No 1.A little 2.Moderately  3.Quite a bit 4. Extremely |
| 5. Paler skin at the swollen limbs | 0.No 1.A little 2.Moderately  3.Quite a bit 4. Extremely |
| 6. Colder skin temperature at the swollen limbs | 0.No 1.A little 2.Moderately  3.Quite a bit 4. Extremely |
| 7. Heated skin temperature at the swollen limbs | 0.No 1.A little 2.Moderately  3.Quite a bit 4. Extremely |
| 8. Swelling at the face and neck | 0.No 1.A little 2.Moderately  3.Quite a bit 4. Extremely |
| 9. Redder skin at the swollen face or neck | 0.No 1.A little 2.Moderately  3.Quite a bit 4. Extremely |
| 10. Paler skin at the swollen face or neck | 0.No 1.A little 2.Moderately  3.Quite a bit 4. Extremely |
| 11. Colder skin temperature at the swollen face or neck | 0.No 1.A little 2.Moderately  3.Quite a bit 4. Extremely |
| 12. Heated skin temperature at the swollen face or neck | 0.No 1.A little 2.Moderately  3.Quite a bit 4. Extremely |
| 13. Please describe the right [radial artery](http://www.baidu.com/link?url=MVSs3fGdvwEA2AKSD8qdoDalhz9oNSm4cVAgQpBP1aFlJz8kJas23aBtbgMGi7guOpOWBTu5jQIcp-ps3u-gqkREHTAyeKS7uzxmixAWMqbZvdQ-8cSBDjUeLqhe67U4) pulse |  |
| 14. Please describe the left [radial artery](http://www.baidu.com/link?url=MVSs3fGdvwEA2AKSD8qdoDalhz9oNSm4cVAgQpBP1aFlJz8kJas23aBtbgMGi7guOpOWBTu5jQIcp-ps3u-gqkREHTAyeKS7uzxmixAWMqbZvdQ-8cSBDjUeLqhe67U4) pulse |  |
